# Subarachnoid hemorrhage-associated brain injury and neurobehavioral deficits are reversed with synthetic adropin treatment through sustained Ser1179 phosphorylation of endothelial nitric oxide synthase

**DOI:** 10.3389/fstro.2024.1371140

**Published:** 2024-03-19

**Authors:** William S. Dodd, Devan Patel, Dimitri Laurent, Brandon Lucke-Wold, Koji Hosaka, Richard D. Johnson, Nohra Chalouhi, Andrew A. Butler, Eduardo Candelario-Jalil, Brian L. Hoh

**Affiliations:** ^1^Department of Neurosurgery, College of Medicine, University of Florida, Gainesville, FL, United States; ^2^Department of Neurosurgery, Jacobs School of Medicine and Biomedical Sciences, University at Buffalo, Buffalo, NY, United States; ^3^Department of Physiological Sciences, College of Veterinary Medicine, University of Florida, Gainesville, FL, United States; ^4^Department of Pharmacology and Physiology and Henry and Amelia Nasrallah Center for Neuroscience, Saint Louis University, St. Louis, MO, United States; ^5^Department of Neuroscience, College of Medicine, University of Florida, Gainesville, FL, United States

**Keywords:** subarachnoid hemorrhage, adropin, eNOS, vasospasm, delayed cerebral ischemia

## Abstract

**Background:**

Subarachnoid hemorrhage (SAH) is a life-threatening vascular condition without satisfactory treatment options. The secreted peptide adropin is highly expressed in the human brain and has neuroprotective effects in brain injury models, including actions involving the cerebrovasculature. Here, we report an endothelial nitric oxide synthase (eNOS)-dependent effect of synthetic adropin treatment that reverses the deleterious effects of SAH.

**Methods:**

We tested the molecular, cellular, and physiological responses of cultured brain microvascular endothelial cells and two mouse models of SAH to treatment using synthetic adropin peptide or vehicle.

**Results:**

SAH decreases adropin expression in cultured brain microvascular endothelial cells and in murine brain tissue. In two validated mouse SAH models, synthetic adropin reduced cerebral edema, preserved tight junction protein expression, and abolished microthrombosis at 1 day post-SAH. Adropin treatment also prevented delayed cerebral vasospasm, decreased neuronal apoptosis, and reduced sensorimotor deficits at seven days post-SAH. Delaying initial treatment of adropin until 24 h post-SAH preserved the beneficial effect of adropin in preventing vasospasm and sensorimotor deficits. Mechanistically, adropin treatment increased eNOS phosphorylation (Ser1179) at 1 & 7 days post-SAH. Treating eNOS^−/−^ mice with adropin failed to prevent vasospasm or behavioral deficits, indicating a requirement of eNOS signaling.

**Conclusions:**

Adropin is an effective treatment for SAH, reducing cerebrovascular injury in both the acute (1 day) and delayed (7 days) phases. These findings establish the potential of adropin or adropin mimetics to improve outcomes following subarachnoid hemorrhage.

## Introduction

The pathophysiology of subarachnoid hemorrhage is complex. Acutely, a sharp rise in intracranial pressure decreases cerebral blood flow and induces transient global ischemia. This early brain injury manifests as cerebral edema, blood-brain barrier (BBB) breakdown, microthrombosis, sympathetic nerve activation, and widespread apoptosis (Geraghty et al., 2019). Over time, the release of cell-free hemoglobin and heme into the subarachnoid space disrupts endothelial cell function by producing reactive oxygen species (ROS) and decreasing nitric oxide (NO) production (Pluta et al., 1996; Pluta, 2005). Diminished vasodilatory tone and activation of vasoconstrictive pathways are hallmarks of the delayed phase of SAH pathology and promote the development of cerebral vasospasm and delayed cerebral ischemia (DCI) (Pluta, 2008).

Cerebral endothelial dysfunction is a critical common element between the pathological mechanisms following subarachnoid hemorrhage (Vergouwen et al., 2008; Peeyush Kumar et al., 2019). From BBB breakdown in the capillary beds to microthrombosis in the small arteries and arterioles, to vasospasm of the large cerebral vessel, all areas of the cerebrovasculature are impacted by SAH. This evidence indicates that treatments exerting a vasoprotective effect will be most efficacious in treating SAH-related disease. Current treatments are only modestly effective (Velat et al., 2011), reaffirming the need for novel therapeutic targets.

Adropin, encoded by the energy homeostasis-associated (*Enho*) gene, was discovered in 2008 by Kumar et al. as a regulator of lipid metabolism and insulin sensitivity, and is highly expressed in the liver and brain tissue of mice (Kumar et al., 2008). Lovren et al. (2010) were the first to demonstrate that adropin has a direct effect on endothelial cell function, increasing VEGFR2 expression and eNOS phosphorylation. Further, they demonstrated adropin improved limb perfusion following hindlimb ischemia. The high levels of endogenous adropin in brain tissue and the direct effect of adropin on endothelium makes adropin a logical candidate for investigation in the context of cerebrovascular disease. Some recent reports demonstrate a protective effect of adropin on the blood-brain barrier, specifically *in vitro* rat brain endothelial cells exposed to simulated ischemia (Yang et al., 2016) and in a mouse model of intracerebral hemorrhage (Yu et al., 2017). We have shown *in vitro* simulation of SAH by exposing endothelial cells to cell-free hemoglobin causes an increase in paracellular permeability, which can be blunted by adropin treatment (Dodd et al., 2021). The precise molecular mechanisms by which adropin mediates these effects remain to be elucidated; however, current literature suggests regulation of nitric oxide bioavailability is an important element. Yang et al. (2018) showed that adropin expression in the brain over the lifespan correlated with eNOS levels and markers of oxidative stress. Given the importance of nitric oxide bioavailability in SAH pathology, we hypothesized that synthetic adropin treatment would improve outcomes following SAH by preventing vascular dysfunction and activating the eNOS pathway.

## Methods

### Animals

All animal experiments were approved by our IACUC (Institutional Animal Care and Use Committee) and performed in accordance with ARRIVE (Animal Research: Reporting of *In Vivo* Experiments) guidelines. All animals were 12–15-week-old females and were housed in pathogen-free housing with *ad libitum* food and water and 12-h light/dark cycling. Wild-type C57Bl/6 mice were received from Charles River Labs (Wilmington, Mass.) and eNOS knockout mice (stock #:002684) were obtained from The Jackson Laboratory (Bar Harbor, Maine). eNOS knockout breeding pairs were genotyped prior to breeding and F1 progeny used for all experiments. The following primers were used for genotyping: eNOS knockout (*Nos3*^−/−^): F - 5′-AGGGGAACAAGCCCAGTAGT-3′, R - 5′-CTTGTCCCCTAGGCACCTCT-3′. All animals underwent twice daily monitoring from institutional animal care services staff and/or investigators. Mice were randomized to treatment groups prior to surgical procedures.

### Pharmaceutics and dosing

Synthetic adropin (Natah et al., 2005; Keep et al., 2012; Vellimana et al., 2017) peptide was supplied from Bachem (Bubendorf, Switzerland) and diluted in 0.1% BSA in saline. 0.1% BSA in saline was used as vehicle control for all experiments. Adropin (450 nmol/kg) or an equivalent volume of vehicle was delivered via intraperitoneal injection 15 min post-SAH. For experiments ending at 24 hours post-SAH, an identical dose of adropin/vehicle was given again 12 h later. For experiments ending 7 days post-SAH, adropin/vehicle was given again on days 1, 3, & 5 post-SAH. In the aneurysm rupture model, adropin was given at a dose of 450 nmol/kg starting 3 days before aneurysm induction (elastase injection). Adropin was given every other day until sacrifice.

### Subarachnoid hemorrhage model

SAH was modeled using the anterior circulation single autologous blood injection method (Sabri et al., 2009, 2011) with minor adjustments. Mice were anesthetized with ketamine (100 mg/kg) and xylazine (10 mg/kg) before removing hair from the scalp and cleaning with serial betadine—saline washes. The tail was also prepped from the base, extending 1 cm distally. Mice were then fixed into a stereotaxic frame, and body temperature was maintained at 37°C. The scalp was incised along the sagittal suture from the bregma to the nasal bone. The skin was reflected, and a burr hole was drilled 5.0 mm rostral of bregma and 0.5 mm right laterally to avoid puncturing dural venous sinuses. Next, 150 μL of arterial blood was drawn from the ventral tail artery onto paraffin wax paper, and hemostasis was achieved with manual pressure using sterile gauze. Fifty μL of blood was drawn into a Hamilton syringe (Hamilton, Reno, Nevada) and passed through the cranial burr hole at an angle of 30° caudally until contact with the skull base (approximately 7.2 mm). The syringe was left in place for 30 seconds to allow for tissue accommodation before blood was manually injected at a rate of 10 μL/minute. After the blood injection was complete, the syringe was left in place for an additional 3 min and then retracted slowly at a rate of 10 mm/minute. Sham surgery was the identical procedure and timing, including placement of the stereotactic needle but without blood injection. After the needle and syringe were removed, the burr hole was covered, and the skin flap was sutured with 5-0 Ethilon suture. Timeline of experiments using SAH model are shown in Supplementary Figure 1.

### Intracranial aneurysm rupture model

Cerebral aneurysms were induced as previously described. Briefly, mice were anesthetized with ketamine (100 mg/kg) and xylazine (10 mg/kg) before the left common carotid artery and the right renal artery were ligated with 7-0 silk suture (Ethicon Inc., Somerville, New Jersey). Carotid and renal artery ligations are hereafter referred to as ligations. One week after ligations, mice were anesthetized and 10 μL of 1.0 U/mL porcine pancreatic elastase solution (Worthington Biochemical Corp, Lakewood, New Jersey) diluted in phosphate-buffered saline (PBS, Invitrogen, Carlsbad, California) was stereotactically injected into the right basal cistern at 1.2 mm rostral of bregma, 0.7 mm lateral of midline, and 5.3 mm deep to the surface of the brain. A subcutaneous osmotic pump (Alzet, Cupertino, California) placed in the right flank immediately after elastase injection and continually infused angiotensin II (Bachem, Torrence, California) at a dose of 1,000 ng/kg/min. Elastase injection and osmotic pump placement are hereafter referred to as aneurysm induction. Following recovery from aneurysm induction, mice were fed a diet of 8% NaCl with 0.12% beta-aminopropionitrile (BAPN; Harlan Laboratories, Indianapolis, Indiana).

Three weeks post-aneurysm induction, mice were deeply anesthetized with ketamine/xylazine. A bilateral anterolateral thoracotomy with transverse sternotomy was made to expose the heart and great vessels. The right atrium was punctured with the tip of a 23 g needle as an outlet before the left ventricle was sequentially perfused with normal saline, 4% paraformaldehyde (PFA), and Coomassie Brilliant Blue dye in a 20% gelatin solution. Brains were carefully removed from the skull and inspected under a dissection microscope for the presence of aneurysms, as evidenced by an outpouching of cerebral arteries in or connected to the circle of Willis. Aneurysm rupture was identified by the presence of extravasated blood near an identified aneurysm. Any mouse exhibiting stroke-like neurological symptoms (decreased motor activity, circling paresis, or ≥15% weight loss) < 3 weeks post-aneurysm induction was immediately euthanized, and brains were inspected for evidence of hemorrhage or aneurysm rupture. Mice that died suddenly prior to the 3-week endpoint were inspected for evidence of intracranial hemorrhage and aneurysm rupture but were not included in histological studies.

### Immunofluorescence

Following perfusion-fixation and overnight post-fixation in 4% PFA, brain samples were embedded in paraffin wax and cut into 0.7 μm sections with a Leica RM2125 RTS microtome (Leica Microsystems, Buffalo Grove, New York) and adhered to poly-L-lysine-coated glass slides. Serial sections were deparaffinized in mixed xylenes (2 × 3 min, 27°C), and rehydrated in graded ethanol solutions and deionized water. Slides were then washed with 1× Tris-buffered saline with 0.05% Tween-20 (TBST) before being incubated overnight with one or more of the following antibodies: rabbit anti-fibrinogen (ThermoFisher). Primary antibodies were detected using donkey anti-rabbit IgG AlexaFluor594, donkey anti-rabbit IgG AlexaFluor488, donkey anti-rat IgG AlexaFluor594, or donkey anti-goat IgG AlexaFluor488 (all from ThermoFisher, Waltham, Massachusetts). FITC-conjugated dUTP-based TUNEL kit was obtained from Abcam (Cambridge, Massachusetts) and performed according to manufacturer's instructions on NeuN-stained sections. Slides were mounted with a DAPI-containing antifade mounting medium (VectaShield, Vector Labs, Burlingame, California) and imaged using an Olympus IX71 fluorescent microscope (Olympus America, Center Valley, Pennsylvania). All images were analyzed by two blinded observers.

### Brain water content measurements

Brain water content was measured using the wet weight—dry weight method (Keep et al., 2012). Twenty-four h post-SAH, brains were removed from the skull, and the olfactory lobe and hindbrain were removed from the cortex and midbrain. Brains were weighed, placed into a 60°C oven, and allowed to dry for 48 h. Dried brain tissue was then reweighed and used to calculate the percent water mass.

### Sodium fluorescein extravasation assay

Sodium fluorescein (NaFlu) extravasation was carried out as previously described (Natah et al., 2005; Saunders et al., 2015). Twenty-four h post-SAH, mice were injected with 100 μL of 2% NaFlu. After 10 min, to allow for full circulation, mice were deeply anesthetized with ketamine/xylazine and perfused with 150 mL of ice-cold PBS to wash out all intravascular fluorescein. Working in the dark, brains were harvested, embedded, and freshly frozen before being cut into 0.7 μm coronal sections onto poly-L-lysine-coated slides. Slides were then mounted and imaged using an Olympus IX71 fluorescent microscope (Olympus America, Center Valley, Pennsylvania). Three images were taken per slide at random locations within the right cerebral cortex. Fluorescein positivity was normalized to a wild-type mouse that did not receive NaFlu injection.

### Cerebral blood flow measurements

One day post-SAH, animals were anesthetized with ketamine/xylazine as described above and placed in a supine position in a stereotaxic frame. The skull was exposed through the longitudinal sagittal incision and cleaned of any debris with a cotton-tipped applicator. A laser speckle contrast imager probe (LSCI, PeriMed, Jarfalla, Sweden) was positioned 10 cm above the skull surface such that the entire skull was in the field of view; the lambdoid suture caudally, frontonasal suture rostrally, and the temporalis muscles laterally. The LSCI sampled blood flow at a rate of 1 sample/second for two continuous minutes. Blood flow (perfusion) values are calculated using PIMsoft software (PeriMed AB, Stockholm, Sweden) as arbitrary “perfusion units.” The value for each animal was the average perfusion over the entire sampling period.

### mRNA collection and qPCR

mRNA was harvested from endothelial cells after 18 h of CFH exposure using a binding column-based kit following the manufacturer-recommended protocol (Cat. #: 74104, Qiagen, Hilden, Germany), and reverse transcription was performed using a kit obtained from New England Biolabs (Cat. #: E6560, Ipswich, MA). cDNA was amplified using a master mix kit (Cat. #: 1725271, BioRad, Hercules, CA) and the following primers: Enho F 5′-ATGGCCTCGTAGGCTTCTTG-3′ and Enho R 5′-GGCAGGCCCAGCAGAGA-3′. Relative cDNA abundance was calculated using the ΔΔCT method.

### Western blotting

At 24 h or 7 days post-SAH, mice were deeply anesthetized and perfused with 10 mL of ice-cold PBS. Brains were harvested from the skull and placed into a cold brain matrix (Harvard Apparatus, Holliston, Massachusetts). The olfactory lobe was removed and brains were cut in half along the mid-sagittal plane. The right hemisphere was cut again 2 mm caudally from the rostral edge and the pieces of tissue were snap frozen in liquid N_2_. Once all tissue samples were collected, brains were homogenized with a Dounce homogenizer and incubated in radioimmunoprecipitation assay (RIPA) buffer at 4°C for 90 min with constant agitation. Samples were centrifuged, supernatant collected, and protein quantified via Bradford assay. Samples were then frozen at −20°C or used immediately. 30 μg of total protein was separated electrophoretically through a 4–15% tris-glycine gel at 90 V for 60 min and transferred onto nitrocellulose membranes with 0.45 μm pore size. Membranes were blocked with 5% skim milk in TBST (5% BSA for phospho-eNOS blots) for 1 h at room temperature and then probed with specified primary antibodies (Table 1) diluted in 5% skim milk (5% BSA for phospho-eNOS) overnight at 4°C with constant agitation. Membranes were then washed 3× with TBST and then incubated with HRP-conjugated secondary antibody diluted in 5% skim milk (5% BSA for phospho-eNOS) for 1 h at room temperature with constant agitation. Finally, membranes were washed 5× with TBST and incubated with luminol-peroxide solution for 45 seconds before being exposed to film. Films were developed and densitometry was performed using ImageJ software.

**Table 1:** Antibodies used in immunoblotting and immunofluorescence experiments.

**Antibody**	**Source**	**Dilution (1 mg/mL stock)**
anti-GAPDH	Abcam (ab8245)	1:10,000
anti-adropin	Cayman chemical (10381)	1:1,000
anti-ZO-1	ThermoFisher (61-7300)	1:2,000
anti-occludin	ThermoFisher (OC-3F10)	1:1,000
anti-MMP9	Sigma (AB19016)	1:2,000
anti-eNOS	Cell signaling technology (9572S)	1:1,000
anti-peNOS(ser1179)	Cell signaling technology (9571S)	1:1,000
Anti-mouse IgG (HRP-conjugated)	Abcam (ab205729)	1:50,000
Anti-rabbit IgG (HRP-conjugated)	Abcam (ab6721)	1:50,000
Rabbit anti-fibrinogen β-chain	ThermoFisher (16747-1-AP)	1:100
Rabbit anti-NeuN	Millipore sigma (ABN78)	1:500

### Delayed cerebral vessel casting

Middle cerebral artery diameter was measured as previously reported (Vellimana et al., 2017; Takemoto et al., 2020). One week post-SAH, mice were deeply anesthetized with ketamine and xylazine before being serially perfused through the left ventricle with PBS (5 mL), 4% PFA (15 mL), and 20% India ink dissolved in 5% gelatin. All solutions were warmed to 37°C to prevent thermic effects on the vasculature and administered at a constant rate of 15 mL/min to mimic cardiac output (Janssen et al., 2002). Mouse carcasses were then stored at 4°C overnight to allow for gelatin hardening. Brains were imaged and analyzed by an observer blinded to group assignment. The narrowest diameter within the first 2,000 μm of the middle cerebral artery (MCA) after the terminal interior carotid artery bifurcation (M1 segment) was measured to assess vasospasm. Mice with anomalous cerebral vasculature (double MCA, bifurcated MCA, etc.) were excluded from data analysis.

### Statistical analysis

All data are presented as mean ± standard error of the mean (SEM) unless otherwise noted. No animals that underwent SAH procedure were excluded from data analysis. Sample size calculations were performed *a priori* using response variables observed in pilot studies, β of 0.20, and α of 0.05. Comparisons of continuous variables between two groups were performed using the Mann-Whitney *U* test or Student's *t* test where appropriate. Non-parametric tests (Kruskal-Wallis with Dunn's multiple comparison) were used to compare differences of continuous variables among three or more groups. For neurobehavioral tests (composite neurological score & corner-turn test), we used a two-way ANOVA with Tukey's multiple comparison's test to determine the effect of group and time. For experiments in the aneurysm rupture model, Fisher's exact test was used to compare proportions of aneurysm formation, rupture, and rupture symptomaticity. Mantel-Cox (Log-rank) test was used to compare symptom-free survival. Power calculations were completed for the delayed vasospasm experiment with an α of 0.05, β of 0.2 to detect a minimum biological difference of 20% between groups and a predicted standard deviation of 15% MCA diameter. All data analysis was performed using Prism data analysis software (GraphPad Software, San Diego, CA).

## Results

### Adropin expression decreases in cultured brain endothelial cells after hemoglobin exposure and in murine brain tissue after SAH

**Figure 1:**
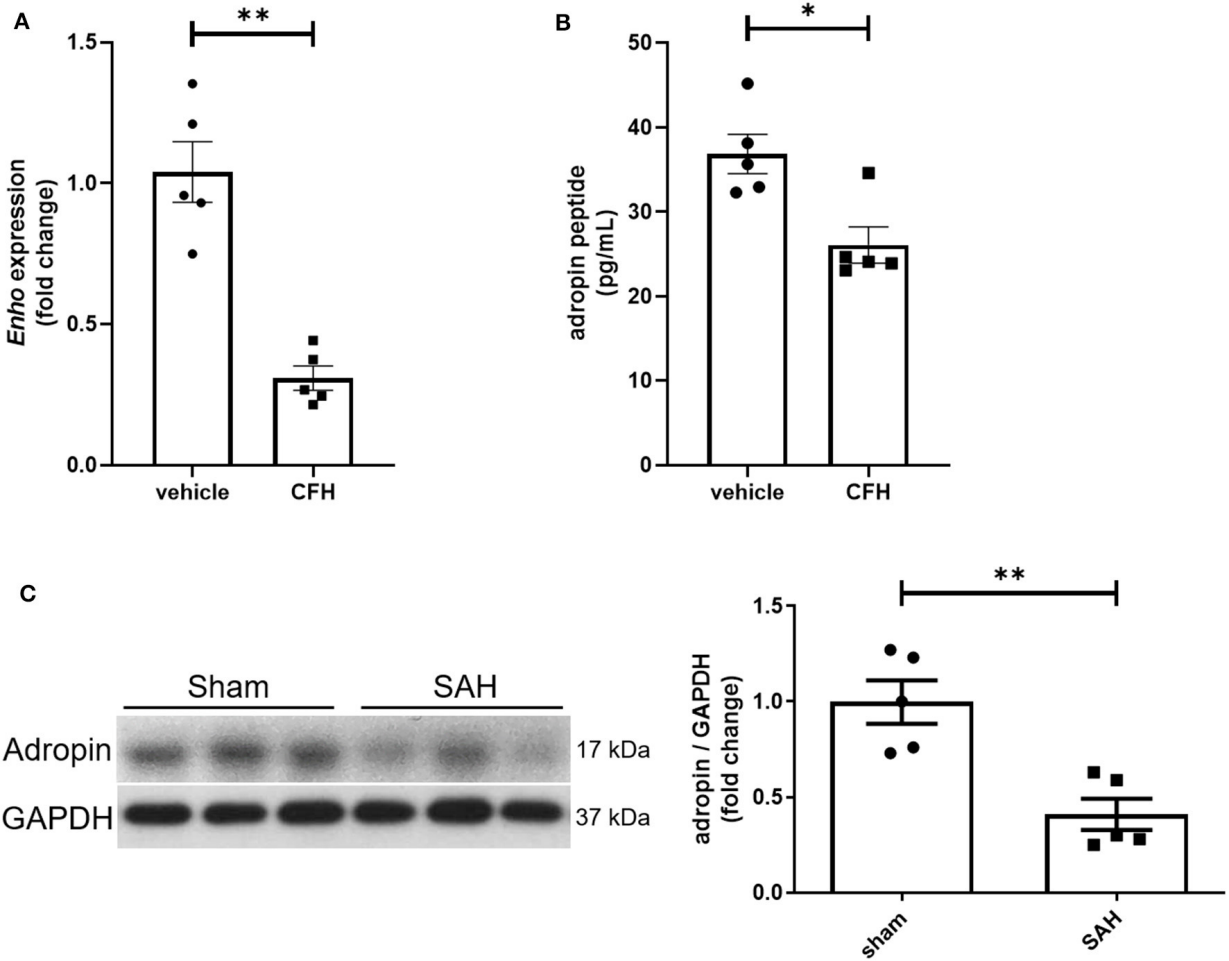
SAH reduces adropin expression. **(A)** Relative mRNA expression in cultured mouse brain microvascular endothelial cells (BMvECs) exposed to cell-free hemoglobin (CFH), **p < 0.01 by Student's *t* test, *n* = 5 each. **(B)** Secreted adropin peptide concentration in BMvEC growth media after CFH exposure, *p < 0.01 by Student's *t* test, *n* = 5 each. **(C)** Western blot of adropin in whole brain lysate in sham (left) and SAH (right) conditions 24 h post-SAH in wild-type mice. Normalized densitometric analysis of adropin immunoblot, **p < 0.01 by Student's *t* test, *n* = 5 each.

While adropin is highly expressed in wild-type mouse brain (Kumar et al., 2008; Yang et al., 2018), the role of disease state in modulating adropin expression has not been well investigated. Given adropin's protective role on endothelium, we hypothesized that decreased adropin expression may be correlated with endothelial dysfunction following SAH. First, we measured transcriptional & translational adropin expression in isolated mouse brain microvascular endothelial cells (BMvECs) after simulated SAH (exposure to cell-free hemoglobin [CFH]). After 18 h of CFH exposure, *Enho* mRNA expression was decreased compared to vehicle-treated cells (1.04 ± 0.11 vs. 0.31 ± 0.04 relative fold change expression) (Figure 1A) as was secreted adropin peptide concentration (36.84 ± 2.34 vs. 26.07 ± 2.15 pg/mL) (Figure 1B). Next, we performed immunoblotting 24 h post-SAH in our mouse model and found that adropin expression is markedly decreased in SAH mice compared to sham (relative expression level: 1.00 ± 0.11 vs. 0.41 ± 0.08) (Figure 1C). Our data suggest endogenous adropin expression falls within 1 day post-SAH.

### Adropin preserves tight junction protein expression and decreases cerebral edema after SAH

**Figure 2:**
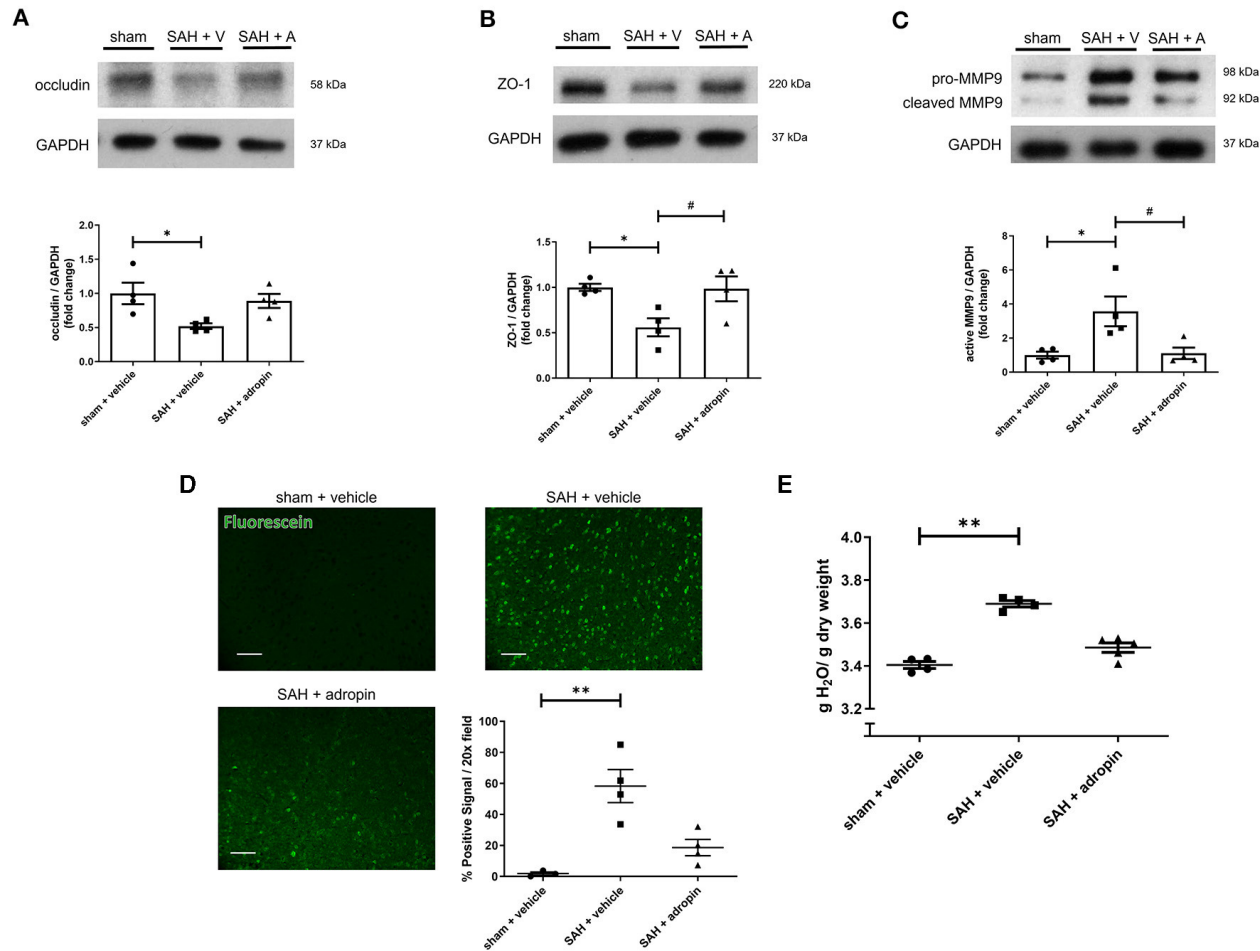
Adropin preserves expression of tight junction proteins and decreases blood-brain barrier permeability. Occludin **(A)**, ZO-1 **(B)**, and MMP9 **(C)** immunoblots in wild-type mice treated with vehicle or adropin, *p < 0.05 compared to sham + vehicle, ^#^*p* < 0.05 compared to SAH + vehicle by non-parametric Kruskal-Wallis test with Dunn's multiple comparisons, *n* = 4 each. **(D)** Blood-brain barrier permeability was measured in wild-type mice by sodium fluorescein extravasation (green) in sham + vehicle, SAH + vehicle, and SAH + adropin groups with semi-quantitative image analysis, **p < 0.01 by non-parametric Kruskal-Wallis test with Dunn's multiple comparisons, *n* = 4 each. **(E)** Cerebral edema (brain water content) after treatment with synthetic adropin peptide, **p < 0.01 by non-parametric Kruskal-Wallis test with Dunn's multiple comparisons, *n* = 4–5 each.

Tight junctions between adjacent endothelial cells are a critical component of the blood-brain barrier and help regulate fluid homeostasis, preventing cerebral edema. Many disease states are characterized by degradation of tight junctions by the protease MMP-9 (Candelario-Jalil et al., 2009; Yang et al., 2019). We investigated the effect of adropin treatment on the tight junction protein occludin, tight junction-associated protein ZO-1, and MMP9 to further elucidate the effect of adropin on barrier function. Treatment with synthetic adropin also preserved the expression of occludin and ZO-1 after SAH compared to control (occludin: sham: 1.00 ± 0.16 relative expression level, SAH + vehicle: 0.52 ± 0.04, SAH + adropin: 0.89 ± 0.10, ZO-1: sham: 0.99 ± 0.04 relative expression level, SAH + vehicle: 0.56 ± 0.10, SAH + adropin: 0.98 ± 0.14) (Figures 2A, B). Consistent with this finding was the observation that SAH increased cleaved MMP9 levels compared to sham, an effect abolished by adropin treatment (sham: 1.00 ± 0.20 relative expression level, SAH + vehicle: 3.57 ± 0.87, SAH + adropin: 1.11 ± 0.34) (Figure 2C). Increased cerebral edema and BBB permeability, important predictors of mortality and neurological outcome, are downstream consequences of tight junction disruption (Claassen et al., 2002; Lublinsky et al., 2019). We hypothesized that adropin would inhibit the development of cerebral edema and reduce BBB permeability. To investigate barrier function, we used a sodium fluorescein extravasation assay. Fluorescein does not bind plasma proteins, making it a sensitive marker for barrier disruption (Saunders et al., 2015). SAH greatly increased barrier permeability as measured by average area of positive fluorescein signal compared to sham (58.32% ± 10.7% vs. 1.8% ± 1.1%) and adropin treatment reduced this effect (18.6% ± 5.2%) (Figure 2D). Similarly, SAH caused an increase in brain water content after SAH and this effect was blunted by adropin treatment (sham + vehicle: 3.40 ± 0.02 g, SAH + vehicle: 3.69 ± 0.01 g, SAH + adropin: 3.49 ± 0.02 H_2_O/g dry tissue) (Figure 2E). These data collectively suggest that adropin treatment prevents disruption of important blood-brain barrier structures and reduces permeability and edema.

### Adropin treatment abolishes microthrombi formation and restores cerebral blood flow after SAH

**Figure 3:**
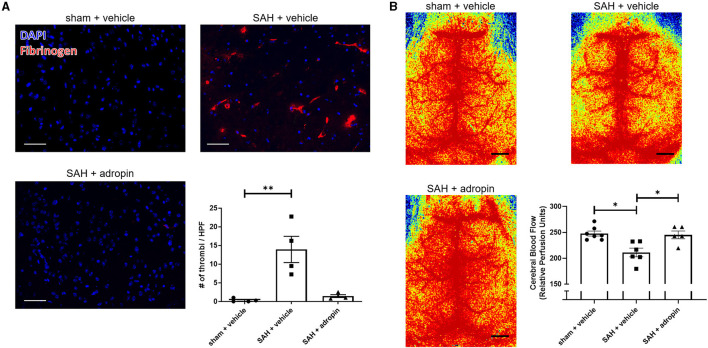
Adropin treatment profoundly reduces microthrombi formation 24 h after SAH. **(A)** Representatives images of fibrinogen- immunostaining (red) in sham + vehicle (top left), SAH + vehicle (top right), and SAH + adropin (bottom left) groups, scale bars = 50 μm, *n* = 4 each, **p < 0.01 by non-parametric Kruskal-Wallis test with Dunn's multiple comparisons. **(B)** Cerebral blood flow on day 1 post-SAH in sham + vehicle (top left), SAH + vehicle (top right), and SAH + adropin (bottom left). *n* = 5–7 per group, *p < 0.05 by non-parametric Kruskal-Wallis test with Dunn's multiple comparisons.

Increased microthrombosis within the cerebral arterioles and capillaries is a contributing factor toward reduced cerebral blood flow and the development of delayed cerebral ischemia (Pennings et al., 2004; Friedrich et al., 2011; Terpolilli et al., 2015). We performed immunostaining for fibrinogen 24 h post-SAH in sham + vehicle, SAH + vehicle, and SAH + adropin mice to evaluate microthrombi formation The SAH + vehicle group had an increased number of microthrombi compared to the sham group (13.9 ± 3.5 thrombi/HPF vs. 0.3 ± 0.2 thrombi/HPF), however adropin treatment completely abolished this effect (1.4 ± 0.4 thrombi/HPF) (Figure 3A). Next, we used laser speckle contrast imaging to quantify cerebral blood flow (CBF) in mice after SAH. SAH induced a significant reduction in perfusion however, adropin treatment restored CBF back to baseline (sham: 247.7 ± 4.85, SAH + vehicle: 211.1 ± 8.34, SAH + adropin: 245.2 ± 7.54 relative perfusion units) (Figure 3B).

### Adropin attenuates delayed cerebral vasospasm and sensorimotor deficits following SAH

**Figure 4:**
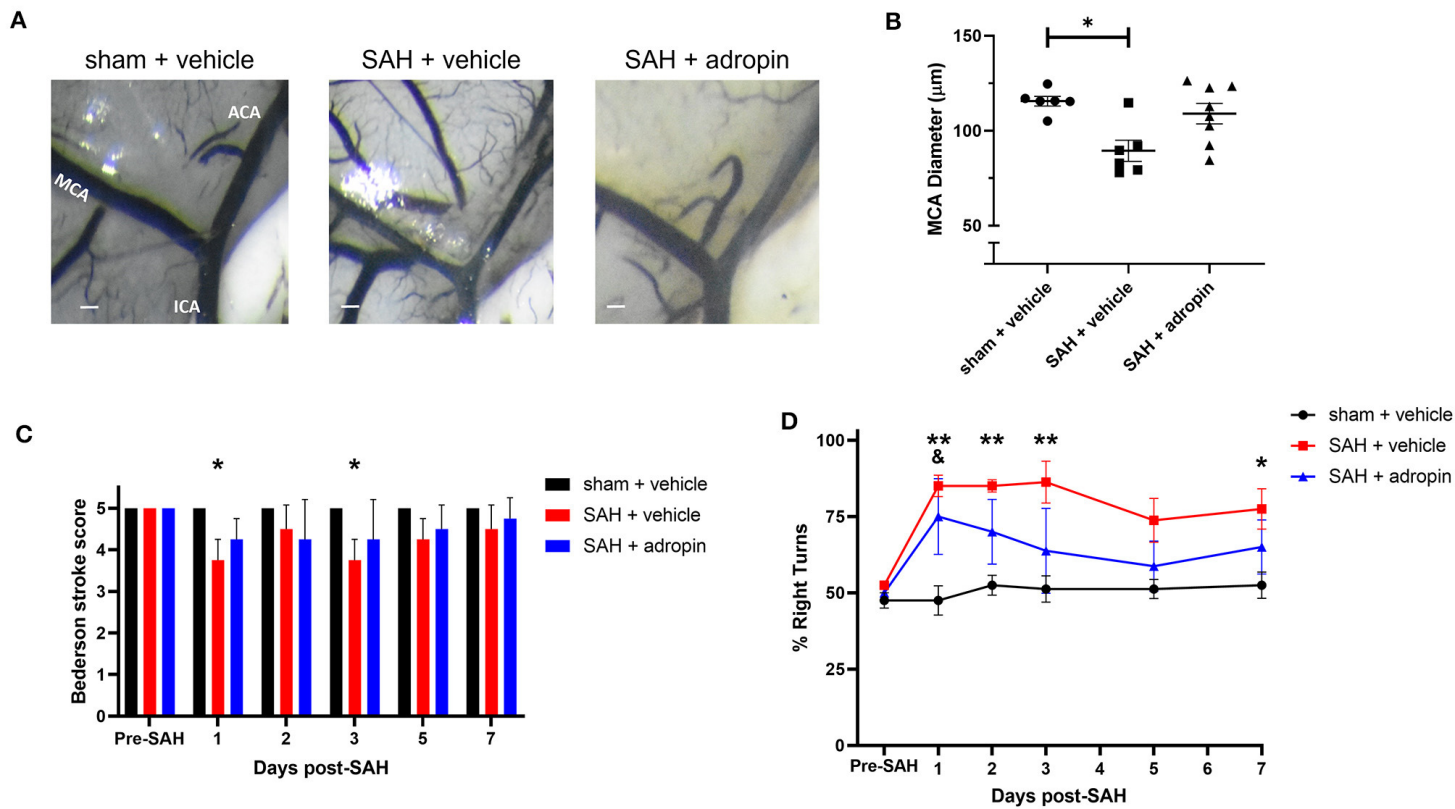
Adropin reduces MCA vasospasm 1-week post-SAH. **(A)** Representative images of dye cast cerebrovasculature one-week post-SAH. Terminal interior carotid (ICA), anterior cerebral (ACA), and middle cerebral (MCA) arteries are labeled in sham images, scale bars = 100 μm. **(B)** Plot of minimum diameter along M1 segment, *p < 0.05 by non-parametric Kruskal-Wallis test with Dunn's multiple comparisons, *n* = 6–8 each. **(C)** Bederson stroke scale scores, *p < 0.05 by ordinary two-way ANOVA with Tukey's multiple comparisons, *n* = 6–8 each. **(D)** Corner-turn preference scores, *p < 0.01 between SAH + vehicle and sham + vehicle groups, **p < 0.01 between SAH + vehicle and sham + vehicle groups, ^&^*p* < 0.05 between SAH + adropin and sham + vehicle groups, *n* = 6–8 each.

Delayed cerebral vasospasm, defined as decreased lumen diameter of the main cerebral vessels, is frequently observed via angiography following SAH (Kassell et al., 1985). As a result, the perfusion to the corresponding cerebrum may be diminished, causing ischemic changed and eventually, functional deficits. We investigated the effect of adropin on cerebral vasospasm by measuring ipsilateral MCA diameter at seven days post-SAH. We observed that SAH + vehicle treatment induced ~20% reduction in lumen diameter compared to sham (100.0 ± 2.2% vs. 77.4 ± 4.8%) and that adropin-treatment reversed this phenotype (94.3 ± 4.7%) (Figures 4A, B). We also analyzed the behavior and functional status of these mice to determine the effect of adropin treatment on the development of functional neurological deficits. We found that our SAH model induces mild neurological dysfunction detected by the Bederson stroke scale (Bederson et al., 1986) (Figure 4C); however, we observed a clear difference in corner turn preference, which is more specific for a deficit created by right-sided SAH and MCA vasospasm. Adropin treatment is capable of reducing this sensorimotor deficit and normalizing corner turn preference (Figure 4D).

### Neuronal apoptosis is reduced by adropin treatment after SAH

**Figure 5:**
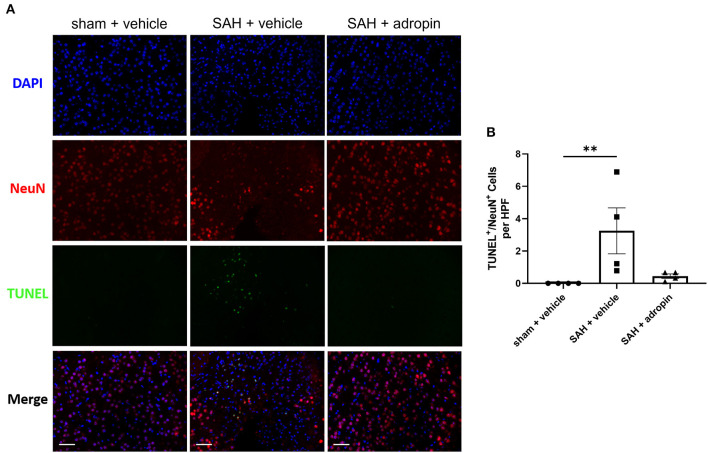
Adropin treatment reduces neuronal apoptosis 1-week post-SAH. **(A)** Representative images of TUNEL staining in sham + vehicle (left), SAH + vehicle (middle, and SAH + adropin (right) groups. Samples were co-stained with NeuN to differentiate between neuronal and non-neuronal cell populations and counterstained with DAPI, 40× magnification (scale bars = 50 μm). **(B)** Quantitative image analysis of TUNEL^+^/NeuN^+^ cells, **p < 0.01 by non-parametric Kruskal-Wallis test with Dunn's multiple comparisons, *n* = 4 each.

Neuronal apoptosis directly contributes to the neurological deficits observed in SAH patients suffering from DCI. To evaluate the effect of adropin on apoptosis in the delayed phase of SAH pathology, we performed TUNEL staining at seven days post-SAH (Figure 5A). An increased number of NeuN^+^/TUNEL^+^ cells were observed in the SAH + vehicle group compared to sham (3.3 ± 1.4 cells/HPF vs. 0.0 ± 0.0 cells/HPF, *p* < 0.01) (Figure 5B). Adropin-treated mice did not display a significant increase in TUNEL^+^ neurons at 1-week post-SAH (0.4 ± 0.3 cells/HPF, *p* > 0.05 compared to sham & SAH + vehicle) (Figure 5B).

### Adropin promotes eNOS pathway activation at 1 and 7-days post-SAH

Nitric oxide is a critical regulator of endothelial function and vasodilation (Frstermann and Mnzel, 2006). eNOS dysfunction after SAH is a well-studied phenomenon and known to contribute to brain injury (Pluta, 2008; Sabri et al., 2011). We investigated the effect of adropin on eNOS expression and activation by performing western blotting against total eNOS and p-eNOS (Ser1179).

**Figure 6:**
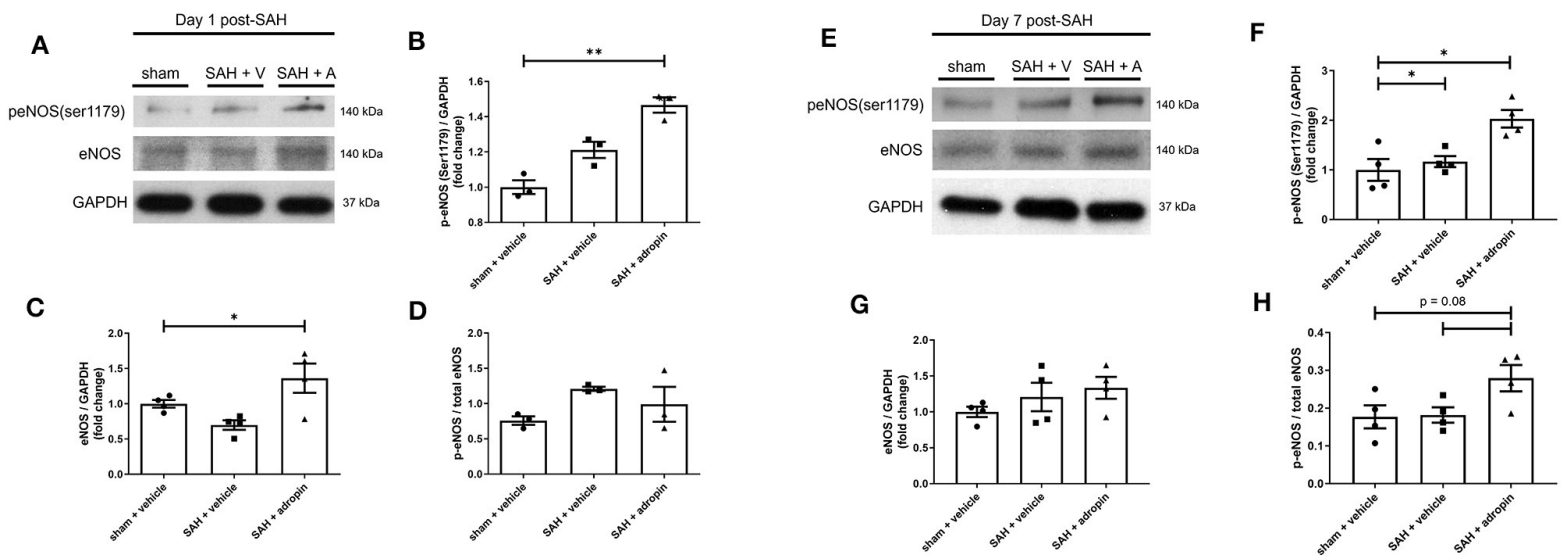
Adropin increases eNOS ser1179 phosphorylation at 1 and 7 days post-SAH. **(A)** Representative immunoblots at 1-day post-SAH. Normalized densitometric values of phospho-eNOS **(B)**, total eNOS **(C)**, and p-eNOS/eNOS ratio **(D)**, *n* = 4 each, * *p* < 0.05, **p < 0.01. **(E)** Representative immunoblots at 7 days post-SAH. Normalized densitometric values for phospho-eNOS **(F)**, total eNOS **(G)**, and p-eNOS/eNOS ratio **(H)**, *n* = 4 each, *p < 0.05.

Treatment with exogenous adropin increased eNOS phosphorylation 1 day post-SAH (1.00 ± 0.04 vs. 1.21 ± 0.05 vs. 1.47 ± 0.04 fold change in sham, vehicle, and adropin groups, respectively) (Figures 6A, B) as well as total eNOS compared to SAH + vehicle (sham: 1.00 ± 0.05 fold change, SAH + vehicle: 0.70 ± 0.07 fold change, and SAH + adropin: 1.36 ± 0.21 fold change) (Figures 6A, C). There was no change in p-eNOS/total eNOS ratios between any groups (Figures 6A, D), likely influenced by the decrease in total eNOS expression in the SAH + vehicle group. Loss of NO bioavailability has been demonstrated to affect both the early and delayed phases of SAH pathology, so we also investigated the eNOS pathway at the 7 day post-SAH timepoint to further delineate adropin's effect on nitric oxide production. Similar to the acute phase, adropin treatment increase phospho-eNOS levels (sham: 1.00 ± 0.22 fold change, SAH + vehicle: 1.17 ± 0.11 fold change, SAH + adropin: 2.03 ± 0.18 fold change) (Figures 6E, F). Total eNOS was also slightly increased in both SAH groups (sham: 1.00 ± 0.07-fold change, SAH + vehicle: 1.21 ± 0.19 fold change, SAH + adropin: 1.34 ± 0.16 fold change) (Figures 6E, G); however, neither reached statistical significance. p-eNOS/eNOS ratio trended higher with adropin treatment as well (sham: 0.18 ± 0.03, SAH + vehicle: 0.18 ± 0.02, SAH + adropin: 0.28 ± 0.03, *p* = 0.08 vs. SAH + vehicle, *p* = 0.08 vs. sham + vehicle) (Figures 6E, H). Our results indicate adropin mediation of the eNOS pathway persists throughout various phases of SAH pathology.

### Adropin has no effect after SAH in mice lacking eNOS

**Figure 7:**
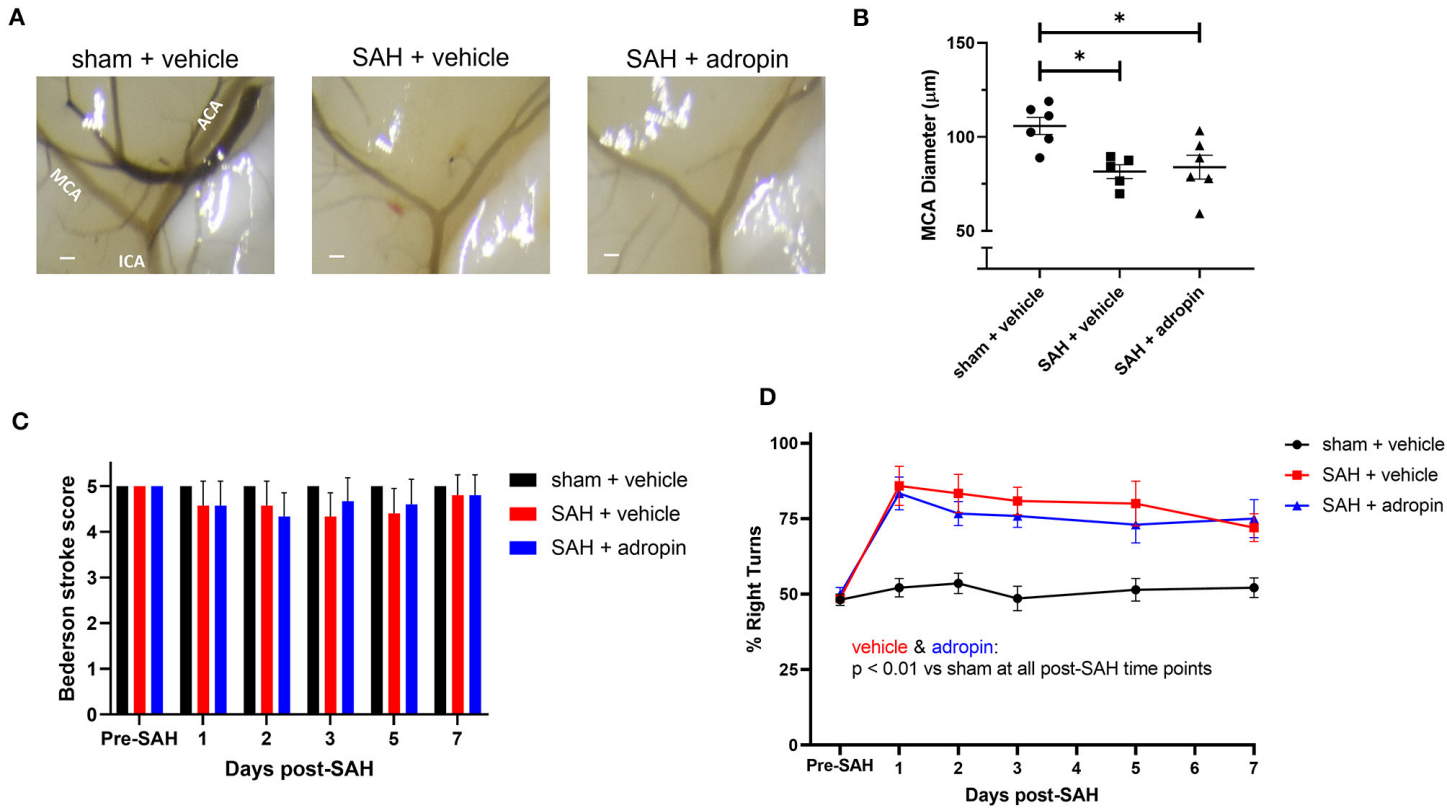
The protective effect of adropin treatment is abolished in eNOS knockout mice. **(A)** Representative images of eNOS^−/−^ mice 1-week post-SAH. Terminal interior carotid (ICA), anterior cerebral (ACA), and middle cerebral (MCA) arteries are labeled in sham images, scale bars = 100 μm. **(B)** Plot of minimum diameter along M1 segment, *p < 0.05 by non-parametric Kruskal-Wallis test with Dunn's multiple comparisons, *n* = 5–6 each. **(C)** Bederson stroke scale scores, *p < 0.05 by ordinary two-way ANOVA with Tukey's multiple comparisons, *n* = 5–6 each. **(D)** Corner-turn preference scores, *p* < 0.01 at all time points between both SAH groups and sham + vehicle group, *n* = 5–6 each.

Given adropin's effects on the eNOS pathway and previous studies of the functional importance of NO signaling after SAH, we hypothesized that adropin's beneficial effect requires the activity of eNOS. To test this hypothesis, we induced SAH in mice with genetic disruption of eNOS (eNOS^−/−^) and randomized them to receive either vehicle or adropin treatment. We found that eNOS^−/−^ mice developed vasospasm similar to wild-type mice and that adropin did not prevent vasospasm (Figures 7A, B). Functionally, adropin also had no effect on sensorimotor deficits. eNOS^−/−^ mice did not develop major deficits as measured by the composite neurological score, but showed increased turn preference in the corner test after SAH regardless of adropin treatment (Figures 7C, D). These findings support the hypothesis that adropin acts through the eNOS pathway to exert its beneficial effects.

### The protective effect of adropin is partially preserved if treatment initiation is delayed until 24 h post-SAH

**Figure 8:**
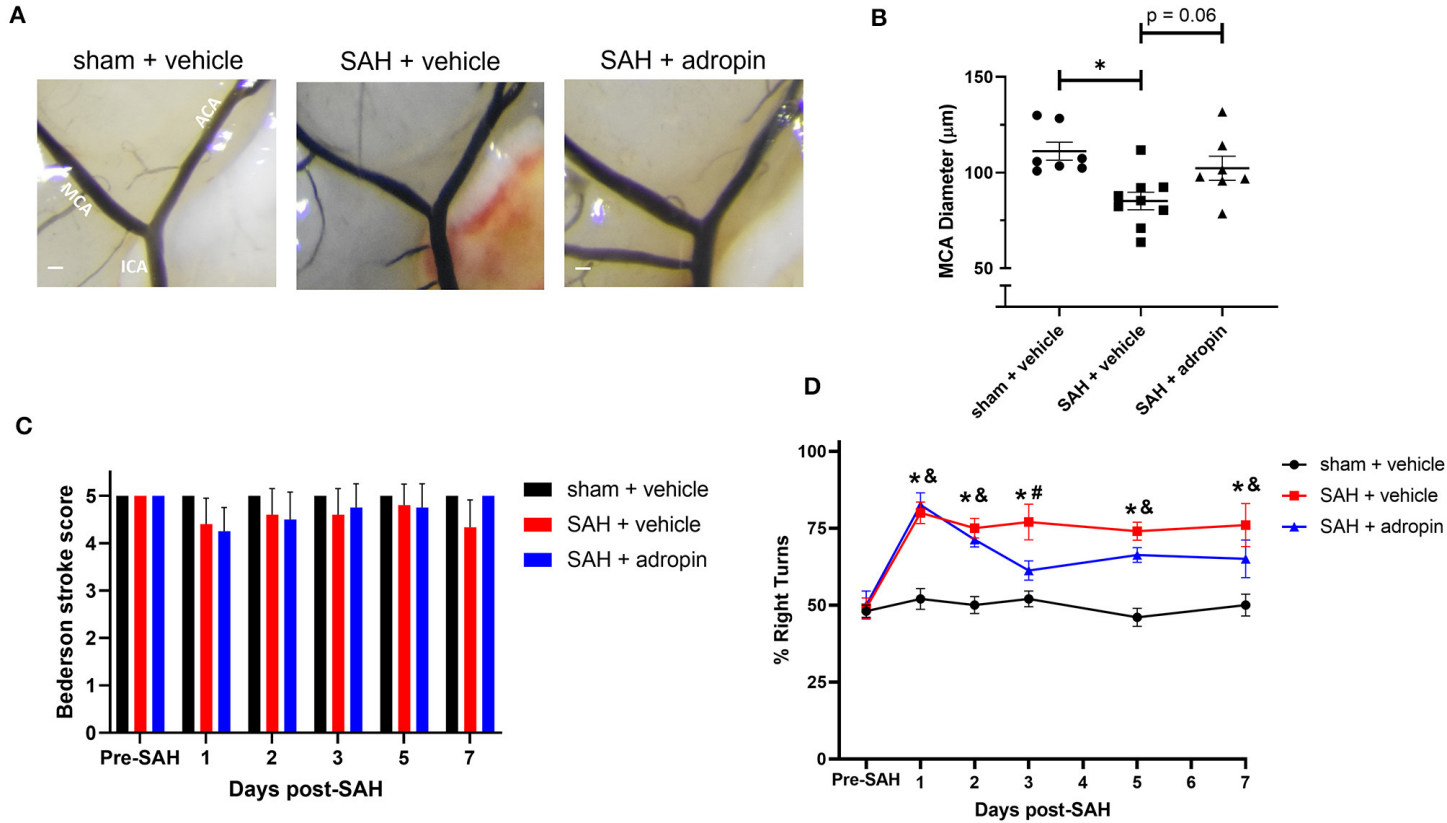
Adropin maintains partial efficacy when treatment is delayed until 24 houst post-SAH. **(A)** Representative images of mice 1-week post-SAH. Terminal interior carotid (ICA), anterior cerebral (ACA), and middle cerebral (MCA) arteries are labeled in sham images, scale bars = 100 μm. **(B)** Plot of minimum diameter along M1 segment, *p < 0.05 by non-parametric Kruskal-Wallis test with Dunn's multiple comparisons, *n* = 7–9 each. **(C)** Bederson stroke scale scores, *p < 0.05 by ordinary two-way ANOVA with Tukey's multiple comparisons, *n* = 7–9 each. **(D)** Corner-turn preference scores, *p < 0.01 between SAH + vehicle and sham + vehicle groups, ^&^*p* < 0.05 between SAH + adropin and sham + vehicle groups, ^#^*p* < 0.05 between SAH + vehicle and SAH + adropin groups, *n* = 7–9 each.

Translational investigation of any therapeutic requires careful consideration of its incorporation into current clinical standards of care. For patients presenting with SAH, the most immediate priority is attaining hemostasis through securing the culprit aneurysm. Treatments to reduce secondary brain injury, especially those which may impact hemodynamics, are likely to be deferred until after surgical/endovascular interventions. We tested the effect of delayed adropin treatment by initiating treatment at 24 h post-SAH instead of 15 min post-SAH. The ability of adropin treatment to improve sensorimotor deficits was maintained under this treatment paradigm, and there was a trend for reduced cerebral vasospasm as well (Figures 8A–D). Separately, there has been some speculation that supraphysiological doses of adropin may be hepatotoxic since adropin is known to regulate liver metabolism. We conducted quantitative assays for common markers of liver toxicity and found that adropin had no effect compared to vehicle treatment (Supplementary Figure 2). These data suggest that adropin could be well-suited for clinical testing and may avoid some of the common pitfalls of novel therapeutics.

### Adropin treatment improves outcomes in murine intracranial aneurysm rupture model

**Figure 9:**
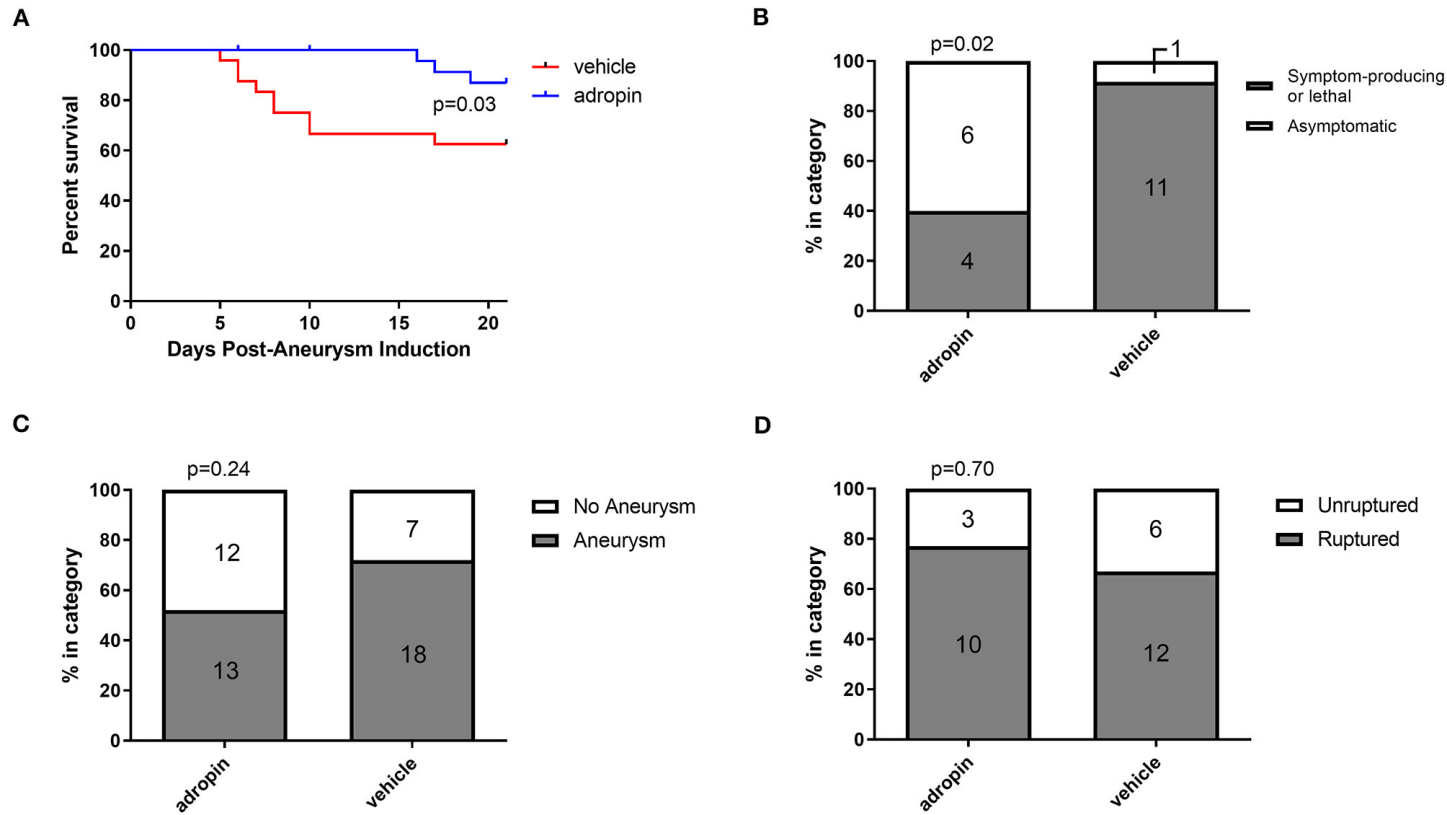
Adropin increases symptom-free survival and decreases rupture symptomaticity following aneurysmal SAH. **(A)** Symptom-free survival curve after aneurysm induction, *p* = 0.03 by Mantel-Cox log-rank test, *n* = 25 at aneurysm induction. **(B)** Proportion of ruptured aneurysms that produced neurological symptoms or death. **(C)** Aneurysm formation rates between vehicle and adropin treatments. **(D)** Aneurysm rupture rates between vehicle and adropin treatments. Data from panels **(B–D)** analyzed with Fisher's exact test.

Given the beneficial effects of adropin in our SAH model, we next sought to test adropin treatment in an aSAH-specific murine model. The presence of aneurysmal change and vascular inflammation prior to subarachnoid hemorrhage distinguishes aneurysmal SAH from other forms of hemorrhagic stroke and SAH. Adropin-treated animals exhibited extended deficit-free survival compared to vehicle control (*p* = 0.02) (Figure 9A). Further, aneurysm ruptures in adropin-treated animals were less likely to be symptom-producing (mice did not exhibit decreased motor activity, weight loss, or circling paresis and evidence of rupture was only found upon autopsy at the end of the study) compared to vehicle-treated animals [4 of 10 (40%) adropin-treated animals became symptomatic vs. 10 of 11 (91%) vehicle-treated animals, *p* = 0.02] (Figure 9B). We found that adropin treatment did not significantly reduce aneurysm formation (13 of 25 adropin-treated animals (52%) vs. 18 of 25 (72%) vehicle-treated animals, *p* = 0.24) (Figure 9C) or aneurysm rupture (10 of 13 adropin-treated animals (77%) vs. 12 of 18 vehicle-treated animals (67%), *p* = 0.70) (Figure 9D). Systemic blood pressure is an important factor in cerebral aneurysm models that could potentially obscure the interpretation of our results. We found no difference in blood pressure between vehicle-treated and adropin-treated mice in our aneurysm rupture model (Supplementary Figure 3).

## Discussion

Our study is the first to define adropin as a protective agent after subarachnoid hemorrhage and among the first to investigate its role in any cerebrovascular disease (Yu et al., 2017). We found that adropin is protective in the early and late phases after SAH. In the early brain injury phase, adropin prevented cerebral edema, reduced BBB damage, and restored cerebral blood flow. In the delayed phase, adropin prevented the development of vasospasm and sensorimotor deficits.

Mechanistically, we found that adropin induces sustained Ser1179 phosphorylation of eNOS (Figures 6B, F), consistent with existing literature (Lovren et al., 2010). The effect of adropin on total eNOS expression is less clear, though it could help preserve eNOS levels in the acute phase (Figure 6C). Our data also show adropin has no effect in mice lacking a functional eNOS enzyme (Figure 7), thus supporting our hypothesis that adropin acts through up-regulation of eNOS signaling. Investigation of the eNOS pathway is often complicated by the tenuous existence of eNOS as a ROS-producing enzyme under oxidative stress conditions, including SAH (Frstermann and Mnzel, 2006; Tejero et al., 2019). The current literature is conflicted on whether the function of eNOS is deleterious after SAH. There is one study that demonstrates eNOS knockout reduces vasospasm by ameliorating eNOS “uncoupling,” the process by which oxidized enzymes produce ROS instead of NO molecules (Sabri et al., 2013). Our data is more consistent with other investigations that have reported eNOS knockout mice develop vasospasm similar to wild-type mice (Vellimana et al., 2011). These findings also provide context to our finding that eNOS phosphorylation is slightly increased in SAH + vehicle conditions 1 week post-SAH. This response could represent an insufficient vasoprotective response to stress or, in eNOS uncoupling environments, could be a contributing factor to vascular injury. Location of SAH and the method of vasospasm induction and measurement may be among the technical factors influencing these results. Further investigation is needed to fully elucidate the causal factors behind these apparent discrepancies.

Another goal of our study was to rigorously evaluate adropin as a preclinical therapeutic. To this end, we sought to introduce clinically relevant variables, including delaying treatment for 24 h and using adropin in a model of cerebral aneurysm rupture. Delayed adropin treatment was still partially effective in reducing corner-turn preference (Figure 8D) and demonstrated a trend in reducing vasospasm (Figure 8B). We believe that these data are proof-of-concept that adropin could be an effective treatment in a realistic clinical setting when treatment can be started sometime within the first 24 h post-SAH. We have also shown that adropin improves survival in a cerebral aneurysm rupture model (Figure 9). These data are harder to interpret due to the inherent variability in aneurysm size, location, degree of hemorrhage, etc.; nonetheless, they demonstrate that adropin can remain effective in a disease model that recapitulates the most common cause of non-traumatic SAH in human patients. In previous studies using our model of aneurysm rupture, we have demonstrated that the highest rate of aneurysm rupture occurs in the 2^nd^ week after aneurysm induction (Hosaka et al., 2014; Hoh et al., 2018; Wajima et al., 2019), consistent with our observations of the vehicle-treated mice in this study. Mice treated with adropin did not show obvious signs of aneurysm rupture but still had identifiable ruptures (gross blood surrounding an aneurysm) upon autopsy at the endpoint of the experiment. We eliminated systemic blood pressure as a possible confounding variable by measuring blood pressure at multiple points before and after aneurysm induction (Supplementary Figure 2).

This study advances the field of SAH research by identifying a novel peptide hormone that has the potential to be used therapeutically; nonetheless, there are limitations that need to be considered. We used female mice in our study, as SAH incidence and mortality are well-documented to be higher in women (Turan et al., 2016); but, there remains the possibility that adropin has sex-dependent effects that need to be elucidated in males. Secondly, our autologous blood injection model has certain advantages (lower mortality, more consistent hemorrhage) over the endovascular puncture model, but is also recognized to be less severe in effect. It is possible that the beneficial properties of adropin would change with increasing severity of SAH.

## Data availability statement

The original contributions presented in the study are included in the article/Supplementary material, further inquiries can be directed to the corresponding author.

## Ethics statement

The animal study was approved by University of Florida Institutional Animal Care and Use Committee. The study was conducted in accordance with the local legislation and institutional requirements.

## Author contributions

WD: Writing—review & editing, Writing—original draft, Visualization, Validation, Project administration, Methodology, Investigation, Funding acquisition, Formal analysis, Data curation, Conceptualization. DP: Writing—review & editing, Methodology, Investigation, Formal analysis, Data curation. DL: Writing—review & editing, Methodology, Investigation, Formal analysis, Data curation. BL-W: Writing—review & editing, Methodology, Investigation, Formal analysis, Data curation. KH: Writing—review & editing, Supervision, Project administration, Methodology, Investigation, Funding acquisition, Formal analysis, Data curation, Conceptualization. RJ: Writing—review & editing, Supervision, Software, Resources, Methodology, Investigation, Formal analysis. NC: Writing—review & editing, Supervision, Software, Resources, Project administration, Methodology, Investigation, Funding acquisition, Formal analysis, Data curation, Conceptualization. AB: Resources, Methodology, Investigation, Funding acquisition, Formal analysis, Conceptualization, Writing—review & editing, Supervision. EC-J: Writing—review & editing, Supervision, Project administration, Methodology, Investigation, Funding acquisition, Formal analysis, Conceptualization. BH: Writing—review & editing, Validation, Supervision, Software, Resources, Project administration, Methodology, Investigation, Funding acquisition, Formal analysis, Conceptualization.

## Supplementary material

Supplementary Figure 1 Experimental timeline. Timeline of SAH model experiments including 24 h (early brain injury) and 7 day (delayed cerebral vasospasm) endpoints. Adropin treatment was initiated at the time of SAH except for Figure 8 wherein it was intentionally delayed to 24 h post-SAH. Behavioral testing was performed at days 0–3, 5, & 7 post-SAH.

Supplementary Figure 2 Adropin treatment is not overtly hepatotoxic. I.P. injection of adropin did not cause elevations in serum concentration of fasting glucose **(top left)**, total bilirubin **(top right)**, aspartate aminotransferase (AST) **(bottom left)**, or alanine aminotransferase (ALT) **(bottom right)**. n = 3 animals for each group.

Supplementary Figure 3 Mean arterial pressure (MAP) of mice before and after intracranial aneurysm induction. Adropin (blue line) and vehicle (red line) treatments had no effect on MAP before or after aneurysm induction, *n* = 8–10 mice in each group at each time point, *p* > 0.99 at all time points.
