# Supplementary figures and images for: Subarachnoid hemorrhage-associated brain injury and neurobehavioral deficits are reversed with synthetic adropin treatment through sustained Ser1179 phosphorylation of endothelial nitric oxide synthase
Source: Front Stroke. 2024 Mar 19;3:1371140. doi: 10.3389/fstro.2024.1371140 (PMC11434178; doi:10.3389/fstro.2024.1371140)

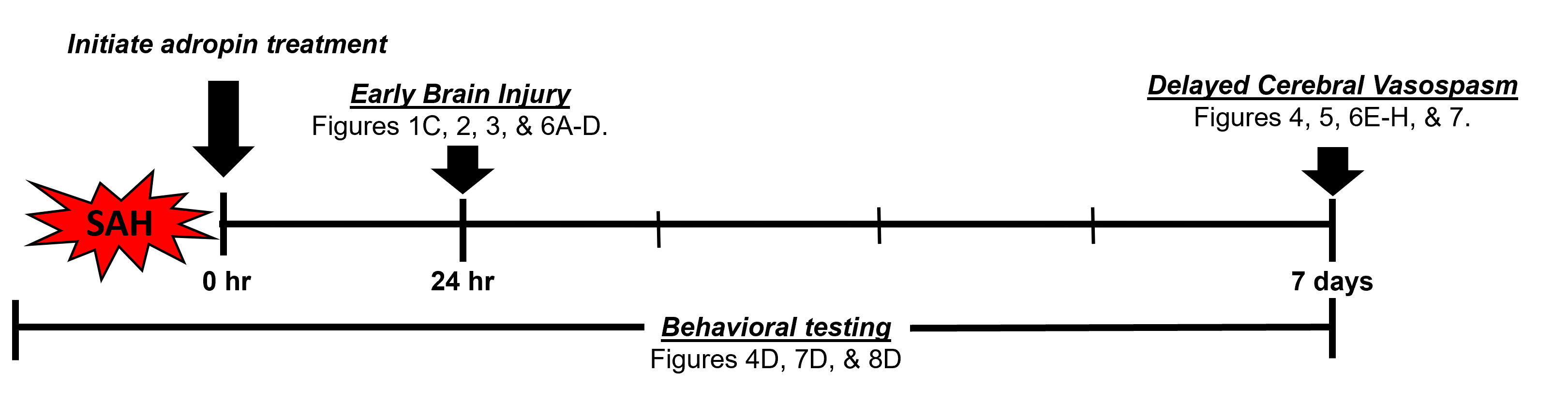

Supplement: Supplementary Figure 1 — Experimental timeline. Timeline of SAH model experiments including 24 h (early brain injury) and 7 day (delayed cerebral vasospasm) endpoints. Adropin treatment was initiated at the time of SAH except for Figure 8 wherein it was intentionally delayed to 24 h post-SAH. Behavioral testing was performed at days 0–3, 5, & 7 post-SAH. [file Image_1.TIF]

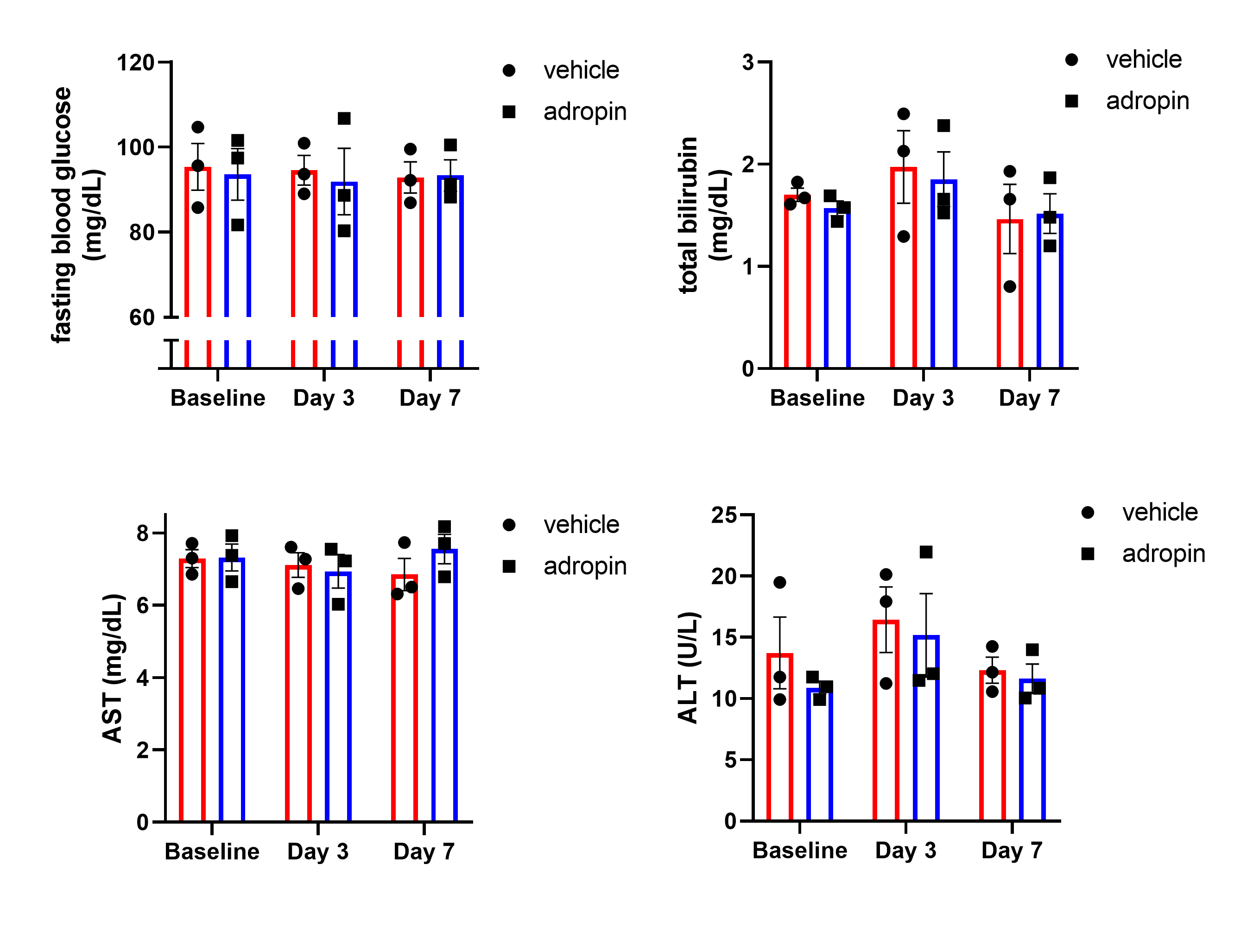

Supplement: Supplementary Figure 2 — Adropin treatment is not overtly hepatotoxic. I.P. injection of adropin did not cause elevations in serum concentration of fasting glucose (top left), total bilirubin (top right), aspartate aminotransferase (AST) (bottom left), or alanine aminotransferase (ALT) (bottom right). n = 3 animals for each group. [file Image_2.TIF]

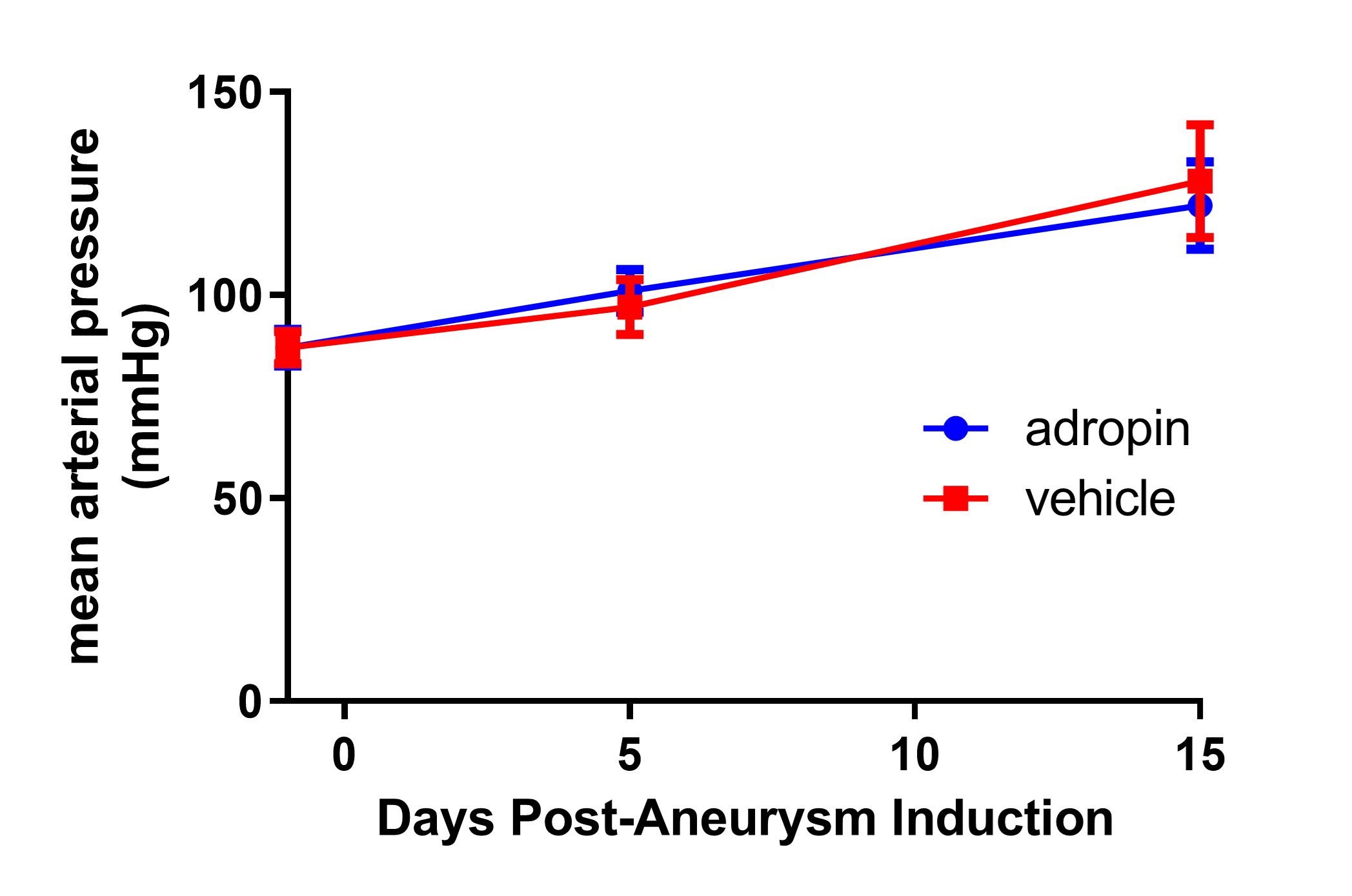

Supplement: Supplementary Figure 3 — Mean arterial pressure (MAP) of mice before and after intracranial aneurysm induction. Adropin (blue line) and vehicle (red line) treatments had no effect on MAP before or after aneurysm induction, n = 8–10 mice in each group at each time point, p > 0.99 at all time points. [file Image_3.TIF]
